# Astrocytoma and IDH1-Wildtype Glioblastoma (GBM) Cells Colonize Tumor Vessels and Deploy Vascular Mimicry

**DOI:** 10.21203/rs.3.rs-2456733/v1

**Published:** 2023-01-17

**Authors:** Haitham H. Maraqah, Mones S. Abu-Asab, Han Sung Lee, Orwa Aboud

**Affiliations:** An-Najah National University; National Eye Institute, National Institutes of Health; University of California Davis; University of California Davis

**Keywords:** Basal lamina, basement membrane, endothelial cells, glioma, pericyte, smooth muscle cell, vessel wall

## Abstract

Gliomas are the most prevalent type of malignant brain tumors with a very dismal prognosis. Angiogenesis in glioma has recently gotten more attention and its molecular aspects have been published; however, these were not complemented with ultrastructural evidence. Our ultrastructural examination of glioma vessels reveals several unique and critical features related to their mechanisms of progression and metastasis strategy. The detailed ultrastructural survey of 18 *IDH1*-wildtype glioblastomas (GBM) and 12 *IDH1*-mutant High-grade gliomas indicated that tumor vessels of both types had undergone deformities such as the thickening of the vessel wall (VW) and proliferation of the basement membrane, contour distortions, abnormal and discontinuous basal lamina, tumor cells’ invasion and colonization of VW, disappearance of endothelial cells (ECs), pericytes, and smooth muscle cells, as well as the formation of a continuous ring of tumor cells attached to the luminal side of VW in numerous cases. The latter feature is a clear sign of vascular mimicry (VM) that was previously suggested in gliomas but never shown by TEM. Additionally, the vascular invasion was carried out by a large number of tumor cells and was accompanied by the accumulation of tumor lipids in the vessels’ lumina and VWs; these two features are distinct for gliomas and may alter the course of the clinical presentation and overall prognosis. This raises the issue of how to specifically target tumor cells involved in vascular invasion in order to optimize prognosis and overcome these mechanisms employed by the tumor cells.

## Introduction

Glioblastoma multiforme (GBM) is an aggressive primary brain tumor with an average survival of around 14 months from the time of initial diagnosis ^1^. Ultrastructural surveys of tumor vessels in gliomas are lacking despite their importance for our understanding of the dynamics of tumor growth and metastasis. Metastasis, the ultimate stage of cancer maturity and the prime cause of mortality, is achieved by gaining entry into the tumor vasculature. The processes of tumor cell intravasation through interaction with the tumor vessels and their endothelium (when present) have been elucidated molecularly but not ultrastructurally. One of the most clinically relevant targeted therapies is tumor neovascularization; this highlights the significance of our investigation.

GBM is considered the most vascularized of all solid tumors with vascular proliferation as their hallmark ^2^, Moreover, the glioma vasculature is the major histological feature that distinguishes GBM from lower-grade gliomas, inevitably results in a potentially more malignant tumor^3^. In spite of this, the status of the vessels in gliomas has not been investigated ultrastructurally. Although our survey shows that some of the abnormal ultrastructural features of tumor vessels are shared by other types of cancers ^4^, we reveal that gliomas vessels have some unique characteristics that could be of clinical relevance. Compared to normal vessels, the tumor vessels are usually large with abnormal morphology and restricted blood flow. The abnormality of tumor vessels has been attributed to their inability to attract and properly organize pericytes ^5^. Normally, pericytes are regularly distributed on the capillaries walls, and in the brain, they play a significant role in vessel generation, blood-brain barrier, immune cell movement, and blood flow ^6^. Tumor vessels may also lack endothelial cells; this highlights the mostly unidirectional flow from the tumors to the bloodstream. The tumor secretions are acidic with lactic acid as the main component. Lactic acid is known to affect neovascularization and vessel structure; this topic has recently become the focus of research ^7^.

Intravasation is a process that may occur in the human body during normal physiological conditions, as illustrated by the entrance of mature thymocytes into the circulation, leukocytes into the bloodstream from zones of inflammation, and dendritic cells into regional lymph nodes ^8^. However, tumor cell intravasation is not a simple penetration where they only go through the vessel wall (VW), reach vessels’ lumina, and start circulating in the bloodstream, it is rather an impressive invasion by a large number of tumor cells that occupy the vessels’ lumina and attach to the luminal side of the VW and appear to replace endothelial cells.

We undertook this ultrastructural study to reveal the architecture of the tumor cell invasion, the physical mechanism of intravasation, and other dynamics applied by glioma tumor cells such as vascular mimicry (VM), basement membrane and its elastic layer proliferation, the efficacy of tumor blood supply, and the presence of tumor cell deposits like lipids in the vessels’ wall and lumia. Additionally, the study allows us to correct some published misconceptions about tumor cell invasion in gliomas.

## Materials And Methods

### Tissue Specimens

All 30 brain tumor tissue specimens were obtained from patients with histopathological confirmed high-grade glioma. The study protocol was approved by the Institutional Review Board of The University of California Davis. Informed consents were obtained from all patients prior to the study. All methods were carried out in accordance with relevant guidelines and regulations. The study utilized specimens of formalin-fixed paraffin-embedded tumors. The specimens represented 12 samples of *IDH1*-mt high-grade glioma tumors and 18 samples of *IDH1*-wt GBMs (Table 1). The tissues were dug out of the paraffin blocks after examining their H&E slides and determining the areas of the tumor viable cells.

### Tissue Preparation

The specimens were deparaffinized in xylene overnight followed by one more change of xylene for 20 min, two changes of absolute ethanol (5 min each), one change of 70% ethanol (5 min), and three changes of phosphate buffered saline (PBS, 5 min each), then processed for transmission electron microscopy (TEM) according to Abu-Asab. Briefly, specimens were washed in PBS, post-fixed in 0.5% osmium tetroxide (OsO_4_), rinsed, dehydrated, then embedded in Spurr’s epoxy resin. Blocks were sectioned at ~ 90 nm thickness on a Leica EM UC6 ultramicrotome (Leica, Austria), double-stained with uranyl acetate and lead citrate, and imaged with JEOL JEM-1010 electron microscope (JEOL, Japan).

## Results

### The Vessels of the IDH1-Wildtype GBMs (Fig. 1):

**Figure 1:**
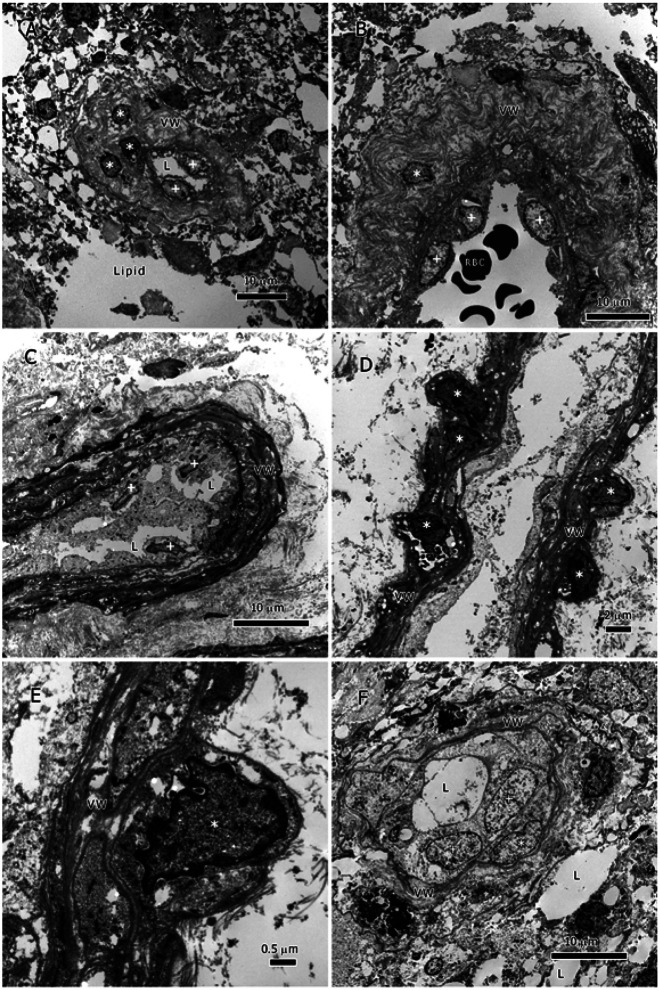
Vessels of the *IDH1*-Wildtype GBMs. All vessels here lack pericytes, smooth muscle-cells, and endothelial cells. The basal lamina is either irregularly discontinuous or absent. A. a small vessel with a thickened VW, lipids (L), and tumor cells in VW (*) and lumen (+); B. part of a large vessel with a very thick VW, and tumor cells in VW (*) and lumen (+); C. partial view of a vessel with a thickened and dense VW, lipids (L), and tumor cells in VW (*) and lumen (+); D. partial view of an elongated vessel with thickened VW, and tumor cells invading the VW (*) and lumen (+); the lumen has cellular debris probably from degenerate endothelial cells; E. a close up of D showing a tumor cell in VW and the details of the VW and the proliferative basement membrane; F. a small vessel surrounded by a proliferative basement membrane showing tumor cells (+) and lipid inclusion (L) in the lumen.

The tumor vessels of the 18 *IDH1*-wildtype GBM tumors showed several abnormal features, some were typical of tumor vessels while others were specific for gliomas. All examined tumor vessels had been invaded and some by a large number of tumor cells. The vessels appeared morphologically distorted having abnormally thickened vessel walls (VW) as well as distorted contours (Fig. 1A-F). In many instances, the VW had enormous thickening that appeared to be due to the chaotic proliferation of the elastic layers of the basement membrane. The thickness of the tumor VW could not be measured due to its high variability within a specimen and between specimens. The basal lamina was distorted, discontinuous, and frequently totally lacking (Fig. 1B-E).

The proliferative elastic layers had gaps between their layers; these gaps were sometimes occupied with lipid inclusions or tumor cells (Fig. 1A-F). The tumor cells appeared embedded in the VW (marked by * in Fig. 1A, B, D-F). Lipid inclusions (L) also were present in the vessels’ lumina (Fig. 1A, C, & F); these inclusions were probably produced by the invading tumor cells or seeped into the vessels from the tumor. Rudimentary remains of endothelial cells were seen on the vessels’ internal side; most of them had vanished and only their debris scattered in the vessels’ lumina (Fig. 1A-E). Occasionally, only tumor cells were seen in the vessels’ lumina or attached to the luminal side (Fig. 1A-C & F), a sign of vascular mimicry (see discussion); erythrocytes were present in vessels (Fig. 1B) but no white blood cells.

### The Vessels of IDH1-Mutant High-Grade Astrocytoma (Fig. 2):

**Figure 2:**
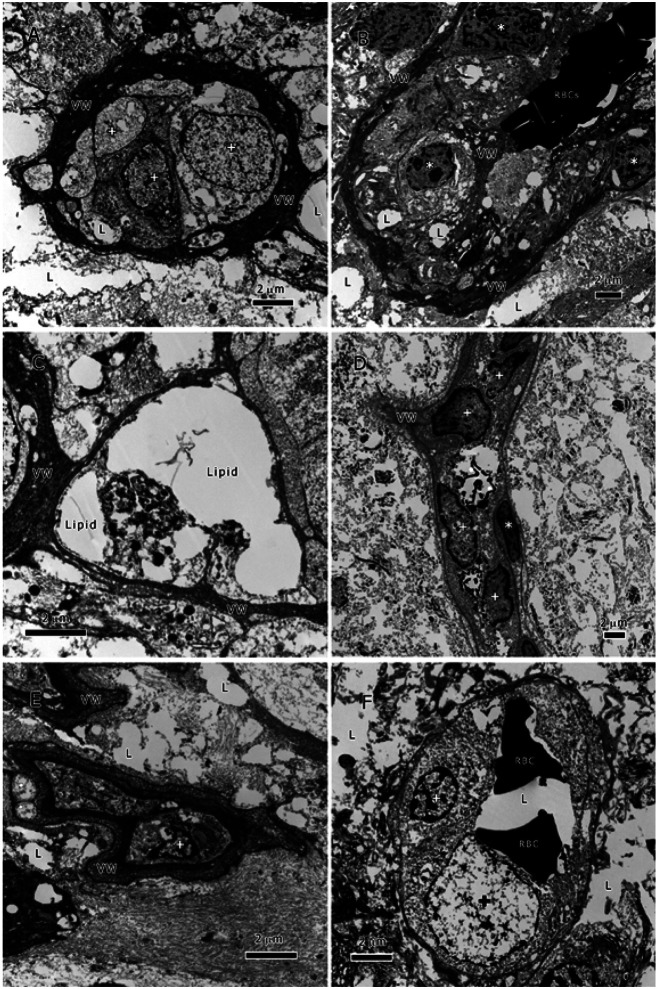
Vessels of *IDH1*-Mutant High-Grade Astrocytoma. All vessels here lack pericytes, smooth muscle-cells, and endothelial cells. The basal lamina is either irregularly discontinuous or absent. A. a small vessel with a thickened and dense VW, as well as lipid (L) and tumor cells (+) in lumen; B. partial view of a large vessel with a slightly thickened VW, lipid inclusions (L) and tumor cells in VW (*), and red blood cells (RBCs) in lumen; C. a vessel with a slightly thickened VW, and lipid inclusions in lumen; D. partial view of an elongated vessel with a redundant basement membrane and tumor cells invading the VW (*) and lumen (+); E. a small deformed vessel with a thickened wall and a tumor cell in the lumen (+); F. a vessel with a proliferative basement membrane showing tumor cells (+), red blood cells (RBC), and lipid (L) in the lumen.

The *IDH1*-mutant astrocytoma tumors showed similar abnormal features in the tumor vessels to that of *IDH1*-wildtype GBM tumors. The tumor cells’ invasion of vessels’ was ubiquitous (Fig. 2) and resulted in deformed and abnormal vessel shape and structure (Fig. 2A-F). The VWs were thickened with redundant elastic layers of the basement membrane, morphologically distorted, and occupied with lipid and tumor cells (*, Fig. 2A-C, & E). The vessels’ lumina had lipid inclusions (L) and tumor cell (+). The basal lamina was abnormal and discontinuous as well as partially or totally lacking. Endothelial cells were absent and replaced by the tumor cells; however, this process is occasionally more prominent with more tumor cells forming a circle around on the luminal side, a sign of vascular mimicry (see discussion). The deformed and distorted structure is more prominent in the mutant type than in wildtype.

It is also worth noting that although the contour morphology as well as VW composition and thickness of the tumor vessels were heterogeneous, some common features transcended their diversity. The vessels of the two cancer types were similar in that they all lacked pericytes, smooth muscle-cells, and endothelial cells; however, they all had tumor cells and lipid inclusions in their VWs and lumina. The amount of tumor cells invading the vessels is staggering and have not been seen in other cancer types that we examined in over the past 25 years.

## Discussion

Angiogenesis is a hallmark phenomenon of solid tumors; it allows new vessels to grow into the tumor to bring in nutrients and at the same time drain lactic acid and other waste products. It also permits the tumor cells to leave the tumor mass and enter blood circulation, and hence, metastasis ensues. Typically, tumor vasculature is a disorganized network of vessels without clear definable arterioles, capillaries, and venules ^9,10^. Tumor vessels have varying diameter and shape with bulges and terminal ends ^11^. Their smooth muscle cells are scarce, endothelium lining discontinuous or absent, and basal lamina abnormal or lacking ^12^.

Our ultrastructural survey of the two types of glioma tumors showed that the tumor vessels have developed unique features that were the result of their interaction with tumor cell invasion and their co-option for the purposes of the tumor cells. These features have implications for aggressive invasion, metastasis, and unfavorable clinical prognosis. The survey has revealed the thickening of the VW and proliferation of the basement membrane, the presence of tumor cells within niches inside VW and their attachment to the luminal side of the VW, as well as the presence of tumor cells and lipid inclusions in the vessels lumina.

The tumor cells of both types had accomplished several tasks that encompassed a successful journeying to and penetration of the vessels as well as the use of the vessels as new colonization sites and conduits for metastasis. It appeared that the endothelial cells (ECs) initial reaction to the tumor was to increase the thickness of the VW, so instead of having a normal thin uniform VW, the tumor vessels had a very thick, pocketed, and proliferative basement membrane. The thick VW did not prevent the tumor cells from reaching the vessels’ lumina as the TEM images have shown (Figs. 1 & 2). Thus, the tumor cells of both glioma types had the capability of invasion and colonization of the tumor vessels as well as the displacement of ECs.

Additionally, glioma cells in the tumor vessels appear to deploy vascular mimicry (VM) once they have colonized the tumor vessels. VM by glioma cells was manifested by their attachment to the luminal side of VWs and tube formation (Figs. 1 & 2). Tumor cells use their β1 integrin subunit to adhere rapidly to the fibronectin of the VW extracellular matrix ^13,14^. VM is a recent concept describing tumor vascular pattern used by many tumors, particularly glioblastoma. The process of VM includes the transition of tumor cells into a vascular phenotype forming tube structure and expressing endothelial surface antigens such as VE-cadherin and EphA2 as well as periodic acid-Schiff (PAS)^15^. The endothelial-like characteristics deployed by the tumor cells ensure their control of the tumor nutrient supply and their metabolic adaptation to resist the immune system assault and anti-angiogenic therapy. Tumor cells with such features are highly adaptable and possess a high metastatic potential. A better understanding of this process will permit the targeting of key steps in VM and provide better treatment options. Our documentation of the ultrastructural characteristics of VM is preliminary and additional research is required to fully elucidate the ontogeny of VM. Therefore, we described here only the pattern of tumor cells’ invasion and colonization of tumor vessels and their abnormal VW.

The enormous thickening of the VW caused by the proliferation of their basement membrane and its elastic layers is a newly observed feature that has not been reported on before. The absence of ECs, pericytes, smooth muscle cells, and sometimes basal lamina is an indication that the VW thickening was most likely carried out earlier before tumor cell invasion and the disappearance of ECs. There are reports that the tumor’s lactic acid could be involved in the loss of ECs due to its damaging action on VE-cadherin ^16^. The events preceding tumor cell invasion that led to the disappearance of the vascular cells are not fully resolved yet and they may take place much earlier during the incipient phases of tumor formation.

Gliomas are highly glycolytic tumors that convert glucose to lactic acid in high amounts to generate ATP and maintain macromolecule synthesis. Thus, in order to survive its own waste product, gliomas efflux lactic acid out of their cells through transmembrane transporters into the tumor microenvironment and drain it through tumor vessels to reduce microenvironment acidification and maintain physiological pH ^17,18^. Lactic acid is known to cause an increased vascular permeability through compromising the integrity of the EC monolayer by loosening their cell-cell adhesion, which subsequently induces the disassembly of the VW components ^16^.

The interaction between tumor cells and ECs rely mainly on VEGF–VEGF receptor signaling ^19^; however, the more detailed aspects of endothelial gene expression in GBM remain unclear^20^, and could vary according to the tumor phases (see below). Vascular targeting by blocking VEGF signaling with bevacizumab has not enhanced overall survival in GBM patients ^21^. This is not surprising since our ultrastructural study showed that ECs were missing from tumor vessels in a much earlier stage and their place was occupied by antiangiogenic therapy-resistant tumor cells that co-opted the vessels for their own benefit. Therefore, our study points out that targeting the VEGF signaling pathway would no longer be effective. Furthermore, understanding the details of vessel invasion and colonization would enable us to target the specific mediators and receptors involved in tumor-EC interaction in order to stop tumor vessel modification and halt tumor cell metastasis.

As we have shown previously, lipid production is a salient feature of gliomas; lipid droplets and inclusions exist within tumor cells and the tumor microenvironment (Maraqah, in press). Lipid production is related to the hypoxic nature of the tumor^22^. In regard to gliomas tumor vessels, we have observed lipid inclusions within the layers of the VW and in the vessels’ lumina. We concluded that the lipid inclusions in the tumor vessels were secreted by the invading tumor cells. Lipids in gliomas pose a serious clinical challenge due to their impact on tumor resistance to therapy. Layers of lipids are barriers to chemotherapy because they limit the entry of non-lipophilic medications and may act as a sink to lipophilic drugs; thus, preventing the medications from reaching the tumor cells in an effective dosage. This tumor feature emphasizes the need for designing lipophilic drugs for gliomas in order to improve drug delivery to tumor cells and achieve treatment success. Also, reducing the amount of lipids in the vessels or the tumor mass could enhance drug delivery. Furthermore, lipid quantitation could be explored as a diagnostic analyte for metastasis or recurrence.

Additionally, we observed that most of the viable tumor cells were located around and inside of the vessels and not inside the bulk of the tumor. No perivascular clasping groups of tumor cells or abluminal tube formations were seen as claimed by some authors ^23^; and no immune cells were visible either. Thus, the added absence of pericytes, smooth muscle cells, and ECs portends the loss of the brain-blood barrier.

In summary, the ultrastructural survey of gliomas has revealed the pattern of tumor cell invasion and colonization of tumor vessels, the tumor-related modifications that vessels undergo, and the pattern of lipid distribution in these vessels. Most of these features were not described before and they could be of clinical implications; therefore, further elucidation of the oncogenic steps of these features and their molecular mechanisms could bring about improved diagnostic and treatment options.

## Figures and Tables

**Table 1:** Demographic of patients, “M” male, “F” female, “WT” wild type, “M” mutant,

Patient #	Sex	Age at Dx yr	IDH status	Location
1	F	59	WT	Frontal
2	F	57	WT	Frontal
3	F	56	WT	Frontal
4	F	55	WT	Thalamic
5	F	26	M	Frontal
6	F	49	M	Frontal
7	M	54	WT	Occipital
8	F	57	M	Frontal
9	M	67	WT	Temporal
10	F	51	WT	Frontal
11	F	30	M	Multifocal
12	F	48	WT	Temporal
13	F	49	M	Frontal
14	M	22	M	Temporal
15	M	39	WT	Frontal
16	M	52	WT	Frontal
17	F	53	WT	Parietal
18	M	44	M	Temporal
19	F	63	WT	Temporal
20	F	35	M	Frontal
21	M	26	M	Frontal
22	M	58	WT	Frontal
23	F	30	M	Frontal
24	F	41	WT	Parietal
25	F	46	WT	Temporal
26	F	28	M	Frontal
27	M	56	WT	Temporal
28	M	50	WT	Frontal
29	M	60	M	Parietal
30	M	43	WT	Frontal

## Abbreviations

EC: Endothelial cell
GBM: glioblastoma
IDH: isocitrate dehydrogenase
IDH-mt: IDH-mutant
IDH1-wt: IDH1-wildtype
VM: vascular mimicry
VW: vascular wall
